# Severe Ketoacidosis Associated with Canagliflozin (Invokana): A Safety Concern

**DOI:** 10.1155/2016/1656182

**Published:** 2016-03-21

**Authors:** Alehegn Gelaye, Abdallah Haidar, Christina Kassab, Syed Kazmi, Prabhat Sinha

**Affiliations:** ^1^Section of Pulmonary and Critical Care Medicine, Providence Hospital and Medical Center, 16001 W 9 Mile Road, Southfield, MI 48075, USA; ^2^Department of Internal Medicine, Providence Hospital and Medical Center, 16001 W 9 Mile Road, Southfield, MI 48075, USA

## Abstract

Canagliflozin (Invokana) is a selective sodium glucose cotransporter-2 (SGLT-2) inhibitor that was first introduced in 2013 for the treatment of type 2 diabetes mellitus (DM). Though not FDA approved yet, its use in type 1 DM has been justified by the fact that its mechanism of action is independent of insulin secretion or action. However, some serious side effects, including severe anion gap metabolic acidosis and euglycemic diabetic ketoacidosis (DKA), have been reported. Prompt identification of the causal association and initiation of appropriate therapy should be instituted for this life threatening condition.

## 1. Introduction

More than 5 million patients are admitted annually to intensive care units (ICUs) in the United States. A number of life threatening medical conditions, including diabetic ketoacidosis, can be associated with metabolic acidosis. Metabolic acidosis may also arise from several drugs and toxins through a variety of mechanisms. Since approval of the first-in-class drug in 2013, data have emerged suggesting that Sodium Glucose Transporter-2 (SGLT-2) inhibitors, including canagliflozin, may lead to diabetic ketoacidosis [1]. We present the case of a 54-year-old male patient who was admitted to the ICU with severe anion gap metabolic acidosis and ketoacidosis with normal glucose levels. All the findings were consistent with canagliflozin (Invokana) induced ketoacidosis.

## 2. Case Description

A 54-year-old Middle-Eastern male with type 1 diabetes mellitus who had laparoscopic appendectomy for acute gangrenous appendicitis with localized peritonitis 2 days prior to his emergency department visit presented with vague chest discomfort, mild abdominal pain, generalized fatigue, and confusion for one day. He denies vomiting, fever, or diarrhea. He had poor appetite and stated not eating and drinking much. He was not paying attention to his carbohydrate count. He denies alcohol or nonprescription drug intake. The patient continued to take his home medication regimen which includes canagliflozin and glargine insulin 60 units at night time. He has been taking canagliflozin 300 mg daily for three years. On presentation, he had a blood glucose level of 142 mg/dL (normal 70–140), normal kidney function (eGFR 103), severe metabolic acidosis with PH of 7.058 (normal 7.35–7.45), anion gap of 37 (normal 8–16) and serum bicarb of 9 mg/dL (normal 22–28), normal lactate level, and *β*-hydroxybutyrate level of 12.4 mmol/L. In the setting of recent abdominal surgery, sepsis with possible diabetes ketoacidosis (DKA) was considered and he was started on intravenous (IV) fluids, IV antibiotics, and insulin infusion along with dextrose 5% in 0.45% NS. However, within few hours of his ICU admission, the patient became more encephalopathic and subsequently intubated. Repeat laboratory test revealed profound anion gap metabolic acidosis with bicarb dropped to 3 mg/dL, elevated serum osmolality of 33 mOsm/kg (normal 275–295), and osmolar gap of 36 (normal 10–15). At this time, he was started on bicarbonate infusion and testing for toxic alcohols, including methanol, ethylene glycol, and diethylene glycol, was performed. Despite the above treatment, he remained acidotic associated with significant electrolyte abnormalities. After nephrology consultation, he was started on fomepizole and to proceed with hemodialysis. Testing for toxic alcohols only showed high levels of acetone (93 mg/dL) and propylene glycol (8.3 mg/dL). Based on the above results, euglycemic ketoacidosis associated with canagliflozin was considered and insulin treatment was intensified until the ketones were cleared while maintaining the serum glucose levels. He required insulin infusion (up to 10 units/hr) along with dextrose to prevent hypoglycemia for 72 hours to normalize the anion gap and clear the ketones. Eventually the patient's symptoms and laboratory data improved leading to successful extubation, toleration of oral diet, and transition to subcutaneous insulin administration with subsequent stable discharge to home.

## 3. Discussion

Sodium Glucose Transporter-2 (SGLT-2) inhibitors, including canagliflozin, dapagliflozin, and empagliflozin, are a newer class of antidiabetic medications that are US Food and Drug Administration (FDA) approved for use with diet and exercise to lower blood sugar in adults with type 2 diabetes. They lower plasma glucose levels by reducing the renal threshold for glucose and increasing urinary glucose excretion. On March 29, 2013, FDA approved canagliflozin (Invokana, Janssen Pharmaceuticals, Inc.), a once-daily tablet indicated as an adjunct to diet and exercise, to improve glycemic control in adults with type 2 diabetes mellitus [2, 3]. Though not FDA approved yet, increasing off-label use of SGLT-2 inhibitors has been observed, most likely due to the favorable insulin-independent glucose-lowering and weight-loss effects.

Results from the preclinical and clinical studies led canagliflozin to be the first-in-class SGLT-2 inhibitor approved in the United States and support canagliflozin as a safe and effective therapeutic option across a broad range of patients with type 2 diabetes mellitus [4]. The comprehensive results of a large phase III clinical development program demonstrate that canagliflozin 100 mg and canagliflozin 300 mg provide substantial and sustained reductions in HbA1c, with additional potentially valuable clinical benefits on BP and body weight [5].

Canagliflozin was generally well tolerated [6]. The most common adverse reactions associated with canagliflozin were genital mycotic infections, urinary tract infections, osmotic diuresis, and reduced intravascular volume. However, rare case reports of diabetic ketoacidosis (DKA) and related events associated with canagliflozin started to occur during the course of treatment. Erondu et al. [7] stated that DKA occurs at a low frequency in 12 patients with type 2 diabetes treated with canagliflozin. Peters et al. [8] also analyzed 13 episodes of SGLT-2 inhibitor-associated euglycemic DKA or ketosis in 9 individuals, seven with type 1 diabetes and two with type 2 diabetes. SGLT-2 inhibitors seem to be associated with euglycemic DKA and ketosis, perhaps as a consequence of their noninsulin-dependent glucose clearance, hyperglucagonemia, and volume depletion. The above findings prompt FDA to investigate the drug. Search of the FDA Adverse Event Reporting System (FAERS) database identified 20 cases of acidosis reported as diabetic ketoacidosis (DKA), ketoacidosis, or ketosis in patients treated with SGLT-2 inhibitors from March 2013 to June 6, 2014. Most of the DKA cases were reported in patients with type 2 diabetes. In most cases, a high anion gap metabolic acidosis accompanied by elevated blood or urine ketones and normal glucose levels was reported. The glucose levels were only mildly elevated at less than 200 mg/dL in some reports. These findings are not typical of diabetic ketoacidosis [1]. The European Medicines Agency had also reported a total of 101 cases of DKA in patients treated with SGLT-2 inhibitors for type 2 worldwide as of 19 May 2015 [9]. According to several studies, the overall frequency of reported events suggestive of DKA is less than 0.1% [7].

Factors identified in some reports as having potentially triggered the ketoacidosis included major illnesses, infections, trauma, reduced food and fluid intake, and reduced insulin dose [1]. Given his reduced oral intake and recent bowel surgery, starvation might have played a role in activating the ketosis pathway in our patient. In addition to high anion gap metabolic acidosis and severe ketoacidosis with low levels of glucose, our patient had an elevated serum osmolal gap, findings generally characteristic of intoxication with one of the toxic alcohols (methanol, ethylene glycol, diethylene glycol, or propylene glycol) [10–12]. However, toxicology study of our patient showed only high levels of acetone and propylene glycol, which was attributed to midazolam infusion.

Unlike in our case, a temporal association with SGLT-2 inhibitor initiation was noted in all cases. The median time to onset of symptoms following initiation of drug therapy was 2 weeks (range 1 to 175 days) [1]. This alerts clinicians to monitor their patients while being on SGLT-2 inhibitors. The potential complications related to SGLT-2 inhibition are predictable, detectable, and preventable so that the balance of benefits and risks favors the use of SGLT-2 inhibitors. Rosenstock and Ferrannini [13] advise that, in any event, type 1 diabetic patients who choose to take this medication off-label should sign an informed consent that makes them fully aware of the potential for euglycemic DKA, the precipitating factors, the warning symptoms and signs, and the preventative measures to adopt.

## 4. Conclusion

Our case clearly illustrates the rare association of severe anion gap metabolic acidosis with ketoacidosis and normal glucose levels with SGLT-2 inhibitors. Even though these patients are euglycemic, they should be treated as if they have diabetic ketoacidosis. Prompt identification of the causal association and initiation of appropriate therapy should be instituted for this life threatening condition. This case also points out that SGLT-2 inhibitors should preferably be avoided in circumstances where poor oral intake is much anticipated.

## Additional Points

Future research should be directed toward identifying which patients are at greatest risk for this side effect. A possible association of SGLT-2 inhibitors and high osmolar gap metabolic acidosis needs to be further investigated.
